# Modulation of leg joint function to produce emulated acceleration during walking and running in humans

**DOI:** 10.1098/rsos.160901

**Published:** 2017-03-08

**Authors:** Dominic James Farris, Brent J. Raiteri

**Affiliations:** 1 School of Human Movement and Nutrition Sciences, The University of Queensland, Brisbane, Australia; 2 Faculty of Sports Science, Ruhr University Bochum, Bochum, Germany

**Keywords:** joint power, mechanical work, leg mechanics, gait, spring, motor

## Abstract

Understanding how humans adapt gait mechanics for a wide variety of locomotor tasks is important for inspiring the design of robotic, prosthetic and wearable assistive devices. We aimed to elicit the mechanical adjustments made to leg joint functions that are required to generate accelerative walking and running, using metrics with direct relevance to device design. Twelve healthy male participants completed constant speed (CS) walking and running and emulated acceleration (ACC) trials on an instrumented treadmill. External force and motion capture data were combined in an inverse dynamics analysis. Ankle, knee and hip joint mechanics were described and compared using angles, moments, powers and normalized functional indexes that described each joint as relatively more: spring, motor, damper or strut-like. To accelerate using a walking gait, the ankle joint was switched from predominantly spring-like to motor-like, while the hip joint was maintained as a motor, with an increase in hip motor-like function. Accelerating while running involved no change in the primary function of any leg joint, but involved high levels of spring and motor-like function at the hip and ankle joints. Mechanical adjustments for ACC walking were achieved primarily via altered limb positioning, but ACC running needed greater joint moments.

## 1. Introduction

Walking and running requires the body's muscle–tendon units (MTUs) to produce forces and absorb energy or generate mechanical work. How the musculoskeletal system achieves this has been studied extensively (for good reviews see [1–4]). Contemporary applications of this research include: increasing understanding of neuromuscular disorders [5,6], probing musculoskeletal injury mechanisms [7,8] and providing bio-inspiration for robotic, prosthetic and wearable assistive technologies [9–14]. It is clear that we need to understand how the human musculoskeletal system generates work for movement, but to date most research has focused on the special case of constant-speed locomotion on level ground (steady-state). However, much of the time we are changing the speed of our locomotion [15], and how the musculoskeletal system adapts to meet the changing work demands of acceleration requires more attention.

Acceleration during locomotion requires net positive work to be done on the body to increase its kinetic energy. Across a range of cursorial species, this is achieved by orienting the ground reaction force (GRF) vector more anteriorly throughout stance, which reduces braking impulse and increases propulsive impulse [16–20]. Dogs and turkeys are particularly effective at this, achieving GRF vector reorientation largely through retraction of their limbs further under their body centre of mass during terminal limb swing [17,19]. Humans are less effective at minimizing braking forces in early stance [21], but similarly retract the limb more prior to ground contact when accelerating compared to constant-speed walking [16] and running [21,22]. An interesting trend observed in humans, turkeys, dogs and wallabies was that positive work at leg joints increased during accelerations via increased joint extension and extension velocities without increased joint moments [16,23–25]. In particular, ankle and hip joint kinematics were adjusted such that net positive work was generated about these joints [19,23–26]. Therefore, work generated at the ankle and hip seems most important for generating net work for acceleration, and understanding how the function of these joints is adapted is important.

Qiao & Jindrich [27] studied human leg joint function during constant-velocity and accelerative walking by characterizing each joint's function in these tasks with functional indexes. These indexes described joint function as being relatively more spring-like, motor-like, strut-like or damper-like with a normalized value for each mechanical function. This approach may be particularly useful for understanding what components a prosthetic or assistive device might need to incorporate. However, Qiao & Jindrich's [27] purpose was only to examine the stance phase of walking. Previous analyses of constant-speed walking have identified sub-phases of the gait cycle incorporating leg swing when the hip stores and returns energy like a spring [28] and when the knee joint exhibits energy absorption/dissipation like a damper [29], the latter inspiring an energy-harvesting knee device [29]. Thus, it would be useful to extend the analyses of [27] to incorporate the swing phase of constant-speed and accelerative walking and to include running. Whole leg [22] and leg joint mechanics [24] have been studied for accelerative running, but not in terms of the indexes presented by Qiao & Jindrich [27].

Therefore, the overarching aim of this study was to examine how lower limb joint function is modulated to produce accelerative walking and running, using functional indexes and detailed analyses of joint kinetics and kinematics. To achieve this, we used our recently evaluated approach for emulating accelerative locomotion mechanics on a treadmill [20]. To further confirm the efficacy of this approach, an initial sub-aim was to verify that this method reproduced the observations of Qiao and Jindrich for walking [27], before extending the analysis to running. We drew the following hypotheses: (i) emulated acceleration conditions on a treadmill would elicit similar adjustments in joint-level function as previously published from overground experiments, and (ii) ankle and hip joints would have high motor indexes during acceleration for both walking and running and this would be achieved predominantly by adjustments to joint angular displacement rather than joint moments. This would require the ankle to change its function from primarily spring-like at constant speed, but the hip would simply remain acting as a motor.

## 2. Methods

### 2.1. Participants and protocol

Ten males (mean ± s.d. age = 26 ± 2 years, height = 179 ± 5 cm, mass = 80 ± 8 kg) gave their written informed consent to participate in this study that was approved by an institutional ethical review committee. Each participant was required to complete four constant-speed (CS) walking trials at 1.25 m s^−1^ and four CS running trials at 2.25 m s^−1^ on a fore-aft split-belt instrumented treadmill (AMTI, USA). Data from the instrumented treadmill was logged at 2000 Hz by QTM software (Qualisys, Sweden) via the motion capture system's A-D board, synchronized to the motion capture marker trajectory data via genlock pulses from the camera system at each camera frame. Each CS trial lasted approximately 30 s and 10 consecutive strides of data were recorded. Each participant also completed 20 emulated acceleration trials (ACC), where the treadmill belt speed was accelerated at 0.76 m s^−2^ from 0.7 m s^−1^ to 2.7 m s^−1^ and the participants naturally transitioned from walking to running. To emulate the inertial forces present in overground accelerations, a posterior horizontal force, equal to the product of body mass and belt acceleration, was applied to their torso via a harness and a tensioned elastic cord, as per a previously detailed method [20]. The force in the elastic cord was measured using a load cell placed in series between the cord and the harness, and sampled at 2000 Hz in the same manner as the treadmill data.

### 2.2. Kinematic modelling and inverse dynamics

An eight-camera motion capture system (Oqus, Qualisys, Sweden) was used to record the three-dimensional positions of reflective markers attached to the pelvis and lower limbs. All marker-position data were filtered with a bidirectional second-order low-pass Butterworth digital filter using a cut-off frequency of 10 Hz and used in conjunction with the rigid body model of Arnold *et al*. [30], modified by removing all segments above the pelvis. Marker positions were recorded during a static calibration trial, where each participant stood in a comfortable stance. These data were used to generate a scaled model for each participant using anatomically positioned markers and standard scaling procedures in OpenSim software v3.0 [31]. Thirty-seven reflective markers were attached to the pelvis and lower limbs of each participant. Twenty of these markers (calibration markers) were placed on strategic anatomical landmarks to define and scale the segments of a seven-segment rigid body model that incorporated the feet, shanks, thighs and a pelvis. For the pelvis, markers were placed over the anterior superior iliac spines (left and right), posterior superior iliac spines (left and right) and the sacrum. The thighs had markers placed over the lateral and medial aspects of the knee joint line and these markers were used with the pelvis markers to scale the thigh. Shank calibration markers included the knee markers and markers placed over the lateral and medial malleoli. For the feet, markers were placed on participant's shoes over the first and fifth metatarsal–phalangeal joints, the calcaneus and the superior aspect of the most distal tip of the toes. The remaining markers were clusters of four markers attached to rigid plates. These rigid plates were strapped to the thigh and shank segments to track the motion of these segments during walking and running. Calibration markers on the feet and pelvis served as tracking markers for those segments. Marker positions were sampled at 200 Hz and logged synchronously with other data in the QTM software (Qualisys, Sweden).

OpenSim software v3.0 was also used to perform an inverse kinematic analysis for each CS or ACC trial. For details see [31], but briefly, this process performs a weighted least-squares fit of the model markers to the experimental marker positions to get the pose of the model at each point in time and from this, joint angles can be determined. We also combined the model kinematics with measured GRF data recorded from two force platforms in the treadmill in an inverse dynamics analysis to compute net muscle moments at the ankle, knee and hip joints using OpenSim v3.0. Before inverse dynamics calculations were implemented, GRF data were low-pass filtered using a bidirectional, second-order, low-pass Butterworth digital filter at a cut-off frequency of 25 Hz. The net joint moments were multiplied by joint velocities (the time derivative of joint angles) to obtain instantaneous joint powers for the ankle, knee and hip. Joint angles were the internal angles between proximal and distal segments with positive changes/velocities indicating extension. Positive joint moments and powers represent moments acting to extend the joint and the rate of work being done to extend the joint, respectively. All data processing following inverse dynamic procedures were conducted in Matlab (The Mathworks, USA) software using custom scripts and functions.

### 2.3. Parsing of emulated acceleration strides

All data were separated into individual strides based on GRF events and stored from each foot–ground contact (GC) to the subsequent ipsilateral GC (i.e. any data from the left leg were stored from left GC to left GC and right limb data from right-to-right). Foot–ground contact was identified as when the vertical ground reaction force for each limb rose through a threshold of 10 N and toe-off as when the vertical force descended through this threshold. Event identification was performed in a custom Matlab GUI, automatically detecting events and allowing for manual deletion of incorrectly identified events. Strides were classified as walking, running or transition, based on the sequence of gait events for that stride. Walking was characterized by a chronological sequence of: foot–ground contact, contralateral toe-off, contralateral foot–ground contact, ipsilateral toe-off and ipsilateral foot–ground contact. Running was characterized by a chronological sequence of: foot–ground contact, ipsilateral toe-off, contralateral foot–ground contact, contralateral toe-off and ipsilateral foot–ground contact. Several additional criteria were enforced to exclude inappropriate strides of data from the ACC trials. First, for walking, any strides where the trailing foot was still in contact with the rear belt (and force platform) when the leading foot moved onto the rear belt were excluded, because right and left leg GRF vectors could not be separately determined. Second, as per previous methods [20], any strides where the overall net work per stride, calculated from external forces (GRF and force in the elastic cord), was less than 90% of the theoretically required value for net work (determined for the participant's body mass and belt acceleration) were removed as being ineffective emulations of overground acceleration (see [20] for details). Third, as there was no CS control to compare walk–run transition strides to, any strides that were not classified as walking or running (i.e. transition strides) based on the unique order of gait events were removed. Fourth, any running strides where the participant's stance foot did not pass from the front treadmill belt to the rear belt in the middle of the stance phase were excluded, because a consistent fore–aft position of the person on the treadmill needed to be maintained [20]. Finally, the stride must have started and been completed while the treadmill belt was accelerating.

### 2.4. Joint functional indexes

To characterize the mechanical function of each joint, we implemented the normalized indexes described by Qiao & Jindrich [27], with a slight modification where the indexes were calculated over entire strides and not just stance. The indexes of strut, spring, motor and damper-like function are described relative to one another as a percentage, with the total adding to 100%. The strut index will typically be high when joint moments are large for prolonged periods but joint work is low (i.e. large moments occur concurrently with minimal joint rotation), and was calculated as follows:

2.1 $$I_{\mathrm{strut}}=\text{max}\left(1-\frac{(t_{\mathrm{HS}}(n+1)-t_{\mathrm{HS}}(n))\int_{\mathrm{HS}(n+1)}^{\mathrm{HS}(n)}|P_{\mathrm{joint}}|\text{d}t}{\int_{\mathrm{HS}(n+1)}^{\mathrm{HS}(n)}|M_{\mathrm{joint}}|\text{d}t},0\right)\times 100\%,$$

where, *I*_strut_—strut index; *t*_HS_—time of heel-strike; *P*_joint_—joint power; *M*_joint_ joint moment. The spring index will be high when periods of negative joint work are immediately succeeded by periods of positive joint work, much like the compression and recoil of a spring. The spring index was calculated as follows:

2.2 $$I_{\mathrm{spring}}=\frac{2\cdot \text{min}(\left|W_{c}^{-}|,|W_{e}^{+}\right|)}{\left|W_{\mathrm{Tot}}^{+}|+|W_{\mathrm{Tot}}^{-}\right|}\times (100\%-I_{\mathrm{strut}}),$$

where, *I*_spring_—spring index; $W_{c}^{-}$—spring-like compression negative work; $W_{e}^{+}$—spring-like extension work; $W_{\mathrm{tot}}^{+}$—total positive work; $W_{\mathrm{tot}}^{-}$—total negative work. Spring-like compression work was defined as negative work absorbed preceding a period of positive power production, such that some or all of the positive power was potentially from the return of stored elastic energy. Where there were multiple periods within a stride that met these criteria, compression work was the sum of all the individual periods of compression work and extension work was the sum of all the individual periods of extension work. The positive work done during positive power after the period of energy absorption was the spring-like extension work. The motor index was determined by

2.3 $$I_{\mathrm{motor}}=\frac{\left|W_{\mathrm{Tot}}^{+}|-\text{min}(\left|W_{c}^{-}|,|W_{e}^{+}\right|\right)}{\left|W_{\mathrm{Tot}}^{+}|+|W_{\mathrm{Tot}}^{-}\right|}\times (100\%-I_{\mathrm{strut}}),$$

where, *I*_motor_—motor index and all other terms are as defined for equation (2.2). The motor index will thus be maximized when large amounts of joint positive work are done without being preceded by similarly large periods of negative joint work. Finally, the damper index primarily represents the extent to which energy is absorbed by negative work at a joint without being potentially returned as positive work by elastic structures. The damper index was calculated as follows:

2.4 $$I_{\mathrm{damp}}=\frac{\left|W_{\mathrm{Tot}}^{-}|-\text{min}(\left|W_{c}^{-}|,|W_{e}^{+}\right|\right)}{\left|W_{\mathrm{Tot}}^{+}|+|W_{\mathrm{Tot}}^{-}\right|}\times (100\%-I_{\mathrm{strut}}),$$

where, *I*_damp_ – damper index and all other terms are as defined for equation (2.2). These indexes provided dimensionless values to compare the function of joints between the CS and ACC conditions and to verify qualitatively that our emulated acceleration induced similar adaptations as overground accelerations have previously been shown to.

### 2.5. Data reduction and statistics

Two participants began the transition to running immediately upon onset of belt acceleration and so could not be included in the analysis of walking. Thus, analyses of running included all 10 participants and analyses of walking had eight participants.

After all outcome variables were determined, time-series data were normalized to 101 points over a stride and the mean waveforms for all participants were used to calculate group means, standard deviations and standard errors for CS walking, CS running, ACC walking and ACC running. All outcome metrics (e.g. functional indexes, joint work values) were similarly averaged. Subsequent statistical tests were run separately on walking and running data. Participant mean joint functional indexes and net joint work values were entered into a two-way [joint × condition (ACC versus CS)] repeated measures ANOVA to test for statistically significant main effects and interactions (*α* = 0.05) using Prism Software (GraphPad Software Inc., USA). *Post hoc* paired *t*-tests with a Bonferroni correction were used to test for pairwise effects. Although reported, joint positive and negative work values were not tested statistically as they are speed-dependent [32] and speed was not controlled.

## 3. Results

**Figure 1.**
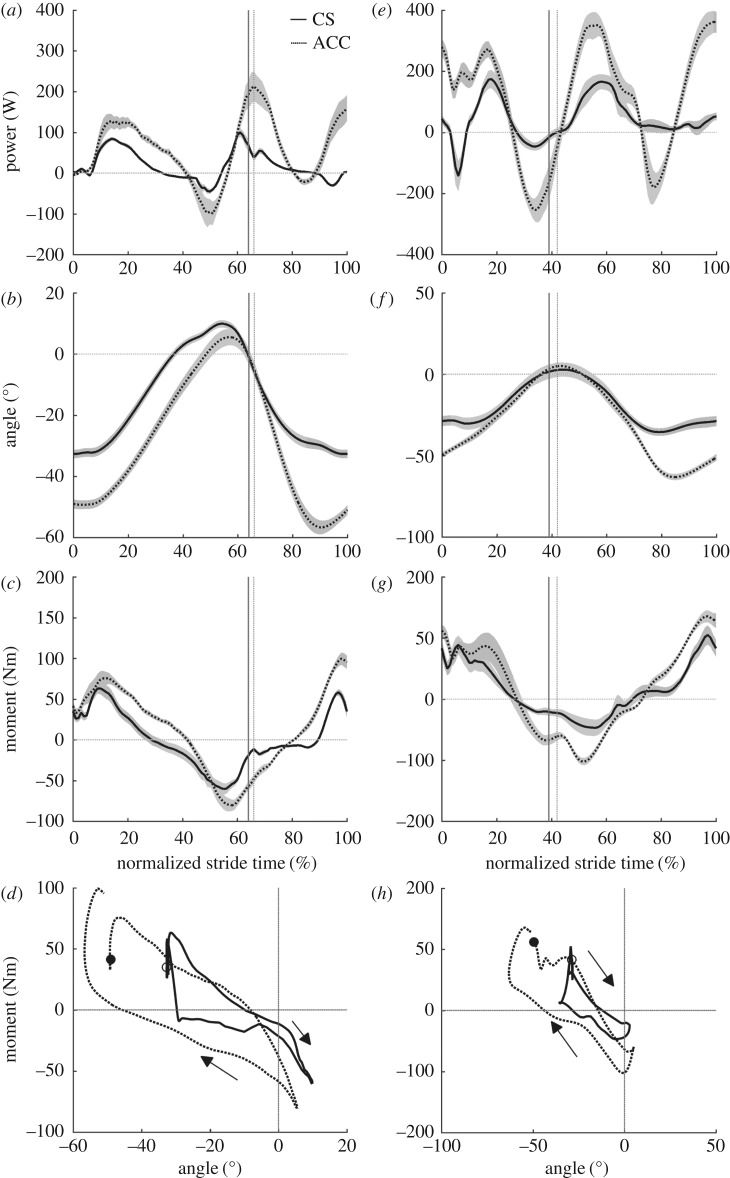
Group mean ± s.e.m. hip joint mechanics for walking (*a–d*) and running (*e–h*). Hip joint power (*a,e*), hip joint angle (*b,f*), hip joint net moment (*c,g*) and hip joint angle–moment plots (*d,h*). Solid lines, CS and dashed, ACC. The vertical lines in panels (*a–c*) and (*e–g*) indicate the end of the stance phase for CS (solid line) and ACC (broken line).

**Figure 2.**
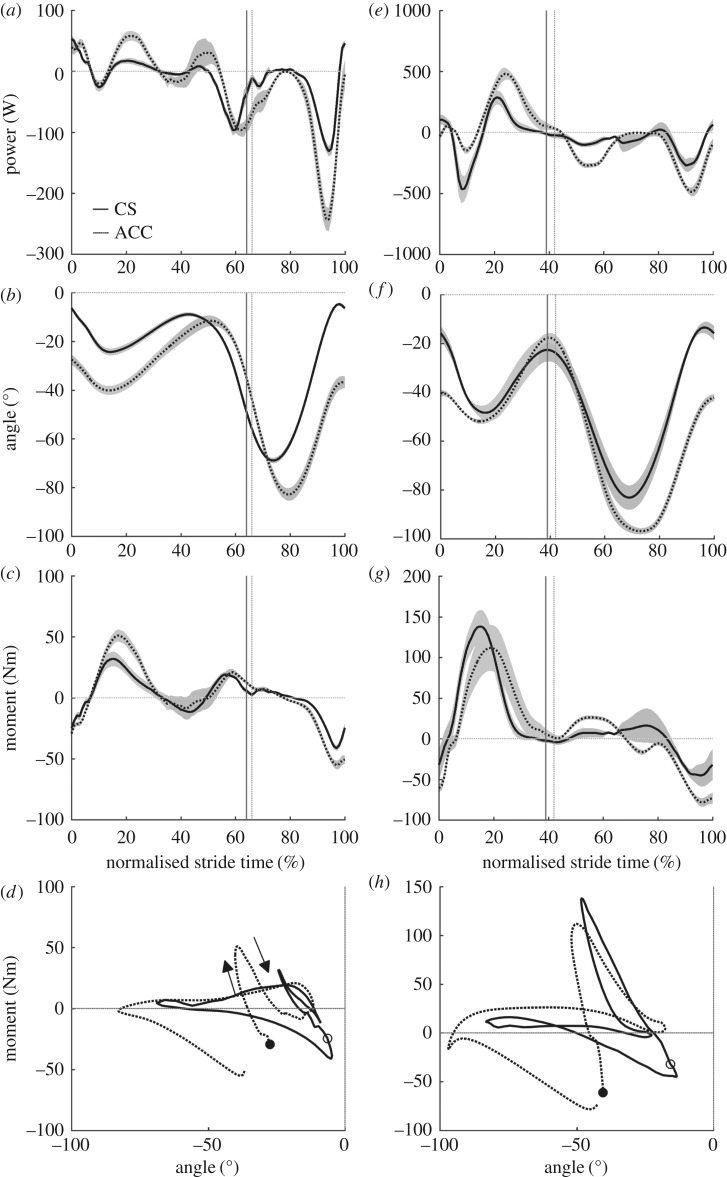
Group mean ± s.e.m. knee joint mechanics for walking (*a–d*) and running (*e–h*). Knee joint power (*a,e*), knee joint angle (*b,f*), knee joint net moment (*c,g*) and knee joint angle–moment plots (*d,h*). Solid lines, CS and dashed, ACC. The vertical lines in panels (*a–c*) and (*e–g*) indicate the end of the stance phase for CS (solid line) and ACC (broken line).

**Figure 3.**
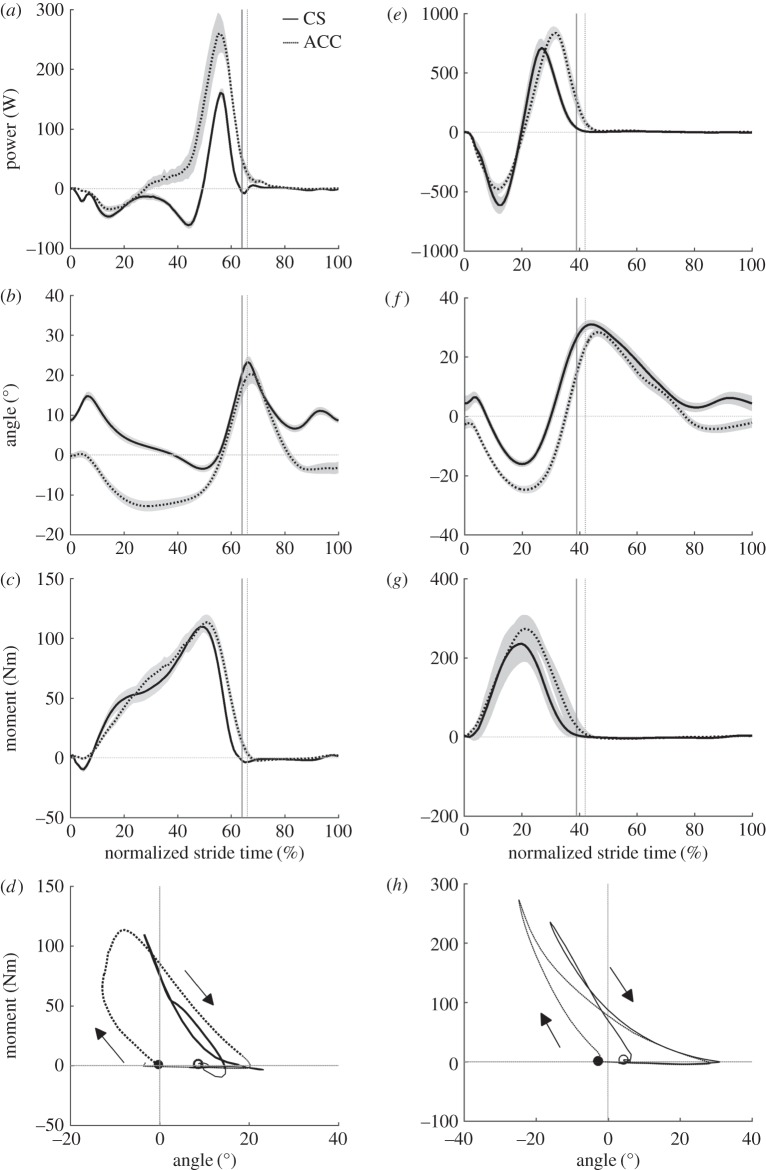
Group mean ± s.e.m. ankle joint mechanics for walking (*a–d*) and running (*e–h*). Ankle joint power (*a,e*), ankle joint angle (*b,f*), ankle joint net moment (*c,g*) and ankle joint angle–moment plots (*d,h*). Solid lines, CS and dashed, ACC. The vertical lines in panels (*a–c*) and (*e–g*) indicate the end of the stance phase for CS (solid line) and ACC (broken line).

There were no differences in average speed between CS and ACC for walking or running (table 1). During CS walking, the hip joint was characterized primarily by motor-like behaviour and its motor index increased for ACC walking (table 2). This increase in motor index was related to a large increase in positive net work at the hip (table 2) that resulted in part from an increase in hip joint extension during stance (ACC = 55 ± 8°, CS = 43 ± 4°, *p* = 0.003; figure 1*b*), but no significant increase in the average hip extension moment during stance phase positive power production (ACC = 44 ± 13 Nm, CS = 34 ± 6 Nm, *p* = 0.1; figure 1*b–d*). During the period of negative hip power in late stance (figure 1*a*), the extension of the hip was not different between CS and ACC, but the average hip moment was more negative (flexion moment) in ACC (ACC = −42 ± 14 Nm, CS = −31 ± 16 Nm, *p* = 0.04; figure 1*b–d*). The knee joint indexes showed that this joint acted primarily like a damper in both CS and ACC conditions, but with a slight shift to motor function for ACC (table 2). This increase in knee joint motor index occurred concurrently with an increase in both average knee joint moment (ACC = 33 ± 11 Nm, CS = 20 ± 10 Nm, *p* = 0.006; figure 2*c*) and knee joint extension during mid-stance (ACC = 29 ± 7°, CS = 16 ± 3°, *p* < 0.001; figure 2*b*). The ankle joint was characterized by predominantly spring-like function during CS walking, but the function of the ankle fundamentally switched to motor-like for ACC walking with its motor index changing from 4% in CS to 67% in ACC (table 2). The switch to motor-index occurred with no concurrent increase in average ankle joint moment during positive power production for ACC walking (ACC = 63 ± 13 Nm, CS = 59 ± 8 Nm, *p* = 0.28; figure 3*c*), but an increase in plantar flexion of the ankle during positive power production (ACC = 36 ± 9°, CS = 27 ± 5°, *p* = 0.004; figure 3*b*). The hip, knee and ankle joints were all more flexed or dorsiflexed at the instant of foot contact with the ground for ACC walking versus CS walking (ankle: ACC = −0.3 ± 2°, CS = 8 ± 2°, *p* < 0.001; knee: ACC = −28 ± 7°, CS = −7 ± 2°, *p* < 0.001; hip: ACC = −49 ± 5°, CS = −33 ± 4°, *p* < 0.001).

**Table 1.** Group mean ± s.d. temporal stride characteristics. (*Significantly different (*p* < 0.05) from CS.)

	walking		running	
	CS	ACC	CS	ACC
speed (m s^−1^)	1.20 ± 0.00	1.24 ± 0.28	2.25 ± 0.00	2.23 ± 0.12
stride time (s)	1.13 ± 0.05	1.33 ± 0.17*	0.78 ± 0.05	0.77 ± 0.08

**Table 2.** Group mean ± s.d. joint mechanical indexes and net mechanical work during constant-speed and accelerative walking and running. (*p < 0.05 for comparison of constant speed with accelerating; *W*_−_ (negative joint work) and *W*_+_ (positive joint work) were not statistically tested. *W*_n_ is net joint work per stride.)

	walking						running					
	CS			ACC			CS			ACC		
	ankle	knee	hip	ankle	knee	hip	ankle	knee	hip	ankle	knee	hip
*I*_spring_	73 ± 19	13 ± 10	30 ± 14	27 ± 9*	6 ± 4	21 ± 11	81 ± 6	31 ± 13	44 ± 11	68 ± 7*	6 ± 7*	45 ± 16
*I*_motor_	4 ± 5	16 ± 7	58 ± 24	67 ± 15*	21 ± 9	75 ± 11*	18 ± 6	14 ± 6	56 ± 11	32 ± 7*	35 ± 9*	55 ± 16
*I*_strut_	18 ± 17	0 ± 0	0 ± 0	6 ± 10	0 ± 0	0 ± 0	0 ± 0	0 ± 0	0 ± 0	0 ± 0	0 ± 0	0 ± 0
*I*_damper_	5 ± 7	71 ± 9	12 ± 15	0 ± 0	73 ± 10	4 ± 2	1 ± 2	55 ± 9	0 ± 0	0 ± 0	59 ± 13*	0 ± 0
*W*_n_(J)	−1 ± 4	−17 ± 5	15 ± 10	26 ± 16*	−25 ± 8*	55 ± 11*	12 ± 22	−37 ± 16	33 ± 13	39 ± 8*	−29 ± 25*	68 ± 9*
*W*_−_ (J)	−15 ± 3	−25 ± 6	−10 ± 10	−7 ± 4	−37 ± 7	−12 ± 8	−51 ± 21	−70 ± 16	12 ± 4	−45 ± 19	−75 ± 14	−32 ± 9
*W*_+_ (J)	14 ± 3	8 ± 3	25 ± 7	35 ± 14	12 ± 7	68 ± 16	63 ± 17	33 ± 9	45 ± 15	84 ± 24	46 ± 16	100 ± 13

During CS and ACC running the functional indexes of the hip joint did not change (table 2). However, the hip joint produced greater positive, negative and net positive work per stride in the ACC running condition compared to CS running (table 2). Positive power was generated about the hip joint during early stance in ACC, when at the same time in CS, negative power was observed (figure 1*e*). This coincided with a more flexed hip position at foot contact in ACC than CS (ACC =−0.3 ± 2°, CS = 8 ± 2°, *p* < 0.001) and an initial period of extension in ACC, when the joint was flexing slightly in CS (approximately the first half of the stance phase, figure 1*f*). The hip joint extended more during stance in the ACC running condition (ACC = 55 ± 7°, CS = 42 ± 8°, *p* = 0.009; figure 1*f*) and had a higher average joint moment during positive power production in stance (ACC = 62 ± 13 Nm, CS = 49 ± 13 Nm, *p* = 0.02; figure 1*g*). Comparing ACC to CS running, the knee became more motor-like and less spring-like (table 2). The knee was also more flexed at foot contact (ACC = −40 ± 4°, CS = −12 ± 3°, *p* < 0.001; figure 1*f*) and flexed less in early to mid-stance (ACC = 12 ± 5°, CS = 36 ± 5°, *p* < 0.001; figure 2*f*). Knee joint extension and average extension moments were not different during stance in CS versus ACC running. The ankle joint was predominantly spring-like in ACC and CS running, although there was a shift to motor-like function and an increase in net positive ankle work in ACC (table 2). The ankle was also more dorsiflexed at foot contact in ACC (ACC = −3 ± 4°, CS = 4 ± 8°, *p* = 0.006; figure 3*f*), but did not dorsiflex or plantar flex by different amounts during stance compared to CS running. There was an increase in average ankle plantar flexion moment during positive power production in stance for ACC (ACC = 118 ± 18 Nm, CS = 98 ± 22 Nm, *p* < 0.001; figure 3*g*).

## 4. Discussion

### 4.1. Modulation of joint function for accelerative walking

The method for emulating accelerative walking and running on a treadmill has previously been shown to recreate the mechanical demands of overground acceleration at the whole-body level [20]. For further affirmation, we qualitatively compared our current findings to a prior study of overground acceleration walking mechanics [27], in terms of functional joint indexes. To allow a fair comparison, we also calculated functional indexes using stance phase data only (table 3) and will refer also to these values in this section. It should be noted that our acceleration in this study (0.76 m s^−2^) was notably higher than that of the study being compared to (0.34 m s^−2^) and thus we would anticipate more exaggerated changes in joint mechanics. Our data showed that, at constant velocity, the hip was predominantly motor-like and its motor index slightly increased for acceleration. This is in agreement with the authors of [27], who showed an increase in hip motor index from 66–73%. In our data, the increase was larger (43–67%, table 3; 58–75%, table 2), which is not surprising given that acceleration was almost double. We found the hip to have generally a much higher spring index than that in [27], which is surprising given the similar looking hip joint power profiles in the two studies (fig. 1A and fig. 2A in [27]). As noted by Shamaei *et al*. [28], the hip is spring-like from terminal stance into early swing, evidenced here by the hip joint moment becoming increasingly negative as the joint extended in late stance and then becoming less negative as the joint flexed in terminal stance and early swing (figure 1*b–d*). In the present study we captured this as spring-like function with spring indexes of 21% or above for all walking data (tables 2 and 3), but it seems Qiao & Jindrich [27] did not, finding a spring index of only 2%. Interestingly, in our data and in [27], the average hip flexion moment during the period of negative power in late stance was more negative for ACC and the amount of joint extension was not different, highlighting the potential for more energy to be stored and returned in elastic structures during accelerative walking. Although the hip flexor musculature does not have highly compliant elastic components, this does not preclude the use of a springs in devices designed to augment hip function and our data suggest that spring-like support could be beneficial during constant speed and accelerative walking.

**Table 3.** Group mean ± s.d. joint mechanical indexes during constant-speed and accelerative walking and running calculated over the stance phase only. (*p < 0.05 for comparison of constant speed with accelerating.)

	walking					
	CS			ACC		
	ankle	knee	hip	ankle	knee	hip
*I*_spring_	73 ± 18	20 ± 14	23 ± 8	27 ± 9*	11 ± 7*	25 ± 10
*I*_motor_	4 ± 5	26 ± 12	43 ± 17	66 ± 15*	41 ± 15*	67 ± 8*
*I*_strut_	18 ± 16	1 ± 4	24 ± 6	7 ± 12*	4 ± 7	3 ± 7*
*I*_damper_	5 ± 7	53 ± 12	10 ± 17	0 ± 0	44 ± 18	5 ± 10

Both our data and that of [27] saw little change in knee function between ACC and CS. Our damper index was higher than in [27] but comparable for stance phase-only data (table 3). Interestingly, the knee damper index was considerably higher for both conditions if the swing phase was included in the calculations (table 2). This is because, in the swing phase, the knee joint exhibits predominantly negative power to decelerate the forward swing of the lower leg (figure 2*a*). Given that the knee joint generates relatively small amounts of positive stance-phase work compared to the other joints (table 2), its role during the swing phase of walking is functionally important and this has been recognized in previous work [29]. However, it does not appear that knee joint function requires any fundamental adjustment for accelerative walking.

Finally, our data agree with [27] regarding the changing role of the ankle for accelerative walking, with the ankle joint switching from predominantly spring-like function for CS to predominantly motor-like function for ACC. Our observed increase in motor index was again greater than in [27], but can probably be attributed to the greater acceleration employed in our protocol. Generally, our results for joint function during emulated acceleration agree well with those of overground data and where there is disagreement, it has been justified above.

### 4.2. Modulation of joint function for accelerative running

We hypothesized that ACC running would require a higher hip joint motor index than CS running. This hypothesis was not supported, despite the hip joint generating both greater positive and net positive work during a stride. Much of the increase in positive hip joint work appeared to come from a period of positive power in early stance in ACC running that was not present in CS running (figure 1*e*). The positive power in early stance for ACC was facilitated by the hip being more flexed at GC and extending throughout stance as opposed to a pattern of flexion and extension that occurred in CS running at the equivalent time. This observation is similar to that of [24]. Surprisingly, however, increasing positive power in early stance did not lead to an increase in the hip joint motor index for ACC running (table 2). The lack of increase in motor index was because ACC running also had a hip joint power profile indicative of potentially spring-like function, with large amounts of negative work occurring in late stance, followed by large amounts of positive work in early swing (figure 1*e*) as a result of a more negative hip joint moment (*p* < 0.001; figure 1*g*) throughout this phase of the stride, with no corresponding difference in joint excursion (*p* = 0.17; figure 1*f*). The net result was that, although more work was generated about the hip joint in ACC, the hip joint remained characterized by an almost equal combination of motor and spring-like function, as it was for CS running (table 2).

The primary function of the knee joint in both ACC and CS running was to act as a damper (table 2). Similar to walking, this was primarily owed to large bursts of negative power as the limb was swinging (figure 2*e*). For ACC running, there was a trade-off of spring-like function for motor-like function (table 2), because the stance phase showed less initial negative power and greater subsequent positive power (figure 2*e*). The former was related to a more flexed knee position at GC followed by less initial knee flexion (figure 2*f*) in ACC, as has previously been observed for overground accelerations [24]. Although the average knee extension moments and knee extension during stance were not different in ACC and CS, the knee extension moment appeared to peak later in ACC, coinciding more with the timing of knee extension and causing a large burst of positive power in the latter half of stance (figure 2*e–g*). Thus, although the knee's primary function remained unchanged for ACC running, adjustments were made to stance-phase mechanics to provide an increase in its motor-like function.

The predominant mechanical function of the ankle during both ACC and CS running was spring-like (table 2). The only change in joint indexes from CS to ACC running was that the motor index increased at the expense of the spring index and this was linked to a threefold increase in net positive ankle joint work (table 2). This increase in net work was owing to greater positive power production in late stance, which was related to an increase in the average ankle joint moment during positive power production, rather than any change in joint excursion. Finding an increased ankle joint moment for ACC running slightly contradicts a prior study [24], where ACC was not linked to increased ankle joint powers or moments. Inspection of figure 3 shows that, in the present data, the increased average ankle moment during positive power production was owing to greater plantar flexion moments being maintained for a longer duration through the stance phase. Thus, the strategy of accelerating runners in our study was to maintain ankle joint moments at a higher magnitude late in the stance phase when the joint was rapidly plantar flexing. This strategy is consistent with observations in accelerating turkeys [17] and in other species running on inclines [33,34], another paradigm where net positive work must be done.

### 4.3. Implications for assistive devices

One application of the data presented here is to provide considerations for how assistive or prosthetic devices might be required to adapt their function or have additional components included to cope with the altered demand of generating accelerative locomotion. In most cases, the principle function of each joint was not required to change for accelerative walking or running, thus any device designed to mimic joint mechanics might not need additional components. However, the control strategies will clearly need to change. A common theme across gaits and joints was that the joint configuration at ground contact was usually different when the body's centre of mass needed to be accelerated versus kept at a constant speed, with joints consistently being more flexed. In running, a key consideration was the time course of joint moments, with the ankle, knee and hip joints all exhibiting joint moments that remained at higher levels during late stance for ACC. This was in contrast to walking, where the ankle and hip had increased motor-like function for ACC, which was achieved primarily via increased joint excursions during positive power production. Thus, initial positioning of the limb may be more important for inducing acceleration in walking, whereas gains on force/torque controls may require adjustment for accelerative running.

Perhaps the most interesting finding was that the ankle joint fundamentally changed function to generate acceleration during walking, shifting from principally spring-like to motor-like (table 2) as net positive work increased for ACC. The angle–moment plot (figure 3*d*) shows that this was not just owing to increased excursion during push-off but also, non-intuitively, to a period of relatively little ankle joint rotation from approximately 30–50% of the stride that halted negative work and opened up the area between loading and unloading in the ankle joint work loop (net positive work). In a study of ankle joint quasi-stiffness [35], this period has been referred to as ‘dual-flexion’ and quasi-stiffness of the ankle during this time was characterized separately from other phases of the stride. It appears from figure 3*d* that the slope of the angle–moment relationship during dual-flexion is steeper in ACC than CS, implying greater quasi-stiffness in ACC. The relationship between ankle quasi-stiffness and acceleration may be worth further investigation from a controls perspective. The ankle's change to motor-like is also interesting when one considers the architecture of the triceps surae muscles that are the prime movers of the ankle joint. These muscles have short pennate fibres that insert through a long compliant tendon. Typically, this architecture is not considered well suited to generating work [2] and is better for behaving in a spring-like manner by cycling energy in the elastic tendon while muscle fascicles contribute relatively little length change [36,37]. Future work should investigate how muscles with such an architecture can be used to help produce net positive work at the ankle joint during accelerative gait.

### 4.4. Limitations

Although it is useful to analyse joint mechanics, the ultimate sources of mechanical work are muscles and their function may look rather different from that of the joints at times. The above discussion of the triceps surae is one example, where the length change of active muscles may be decoupled from the rotation of the joint(s) they span. Furthermore, biarticular muscles act across two joints and could have rather different mechanical functions from either joint. For example, in walking, during late stance, the ankle plantar flexes and generates positive power while the knee flexes and absorbs energy. These actions have counteracting effects on the biarticular gastrocnemius muscles, which may not change length and act as a strut, spanning both joints. This example highlights that if one seeks to understand individual muscle mechanics contributing to acceleration, or indeed develop assistive devices based on precise replication of biological muscle function, one needs to study individual muscle functions throughout the entire limb and how their coordinated actions allow power to be transferred from muscles situated proximally in the limb to distal joints.

The treadmill-based emulation of acceleration was not a perfect recreation of acceleration dynamics. While we have shown previously [20] and in this paper that the method induces appropriate mechanics to approximate overground accelerations, applying a horizontal force approximately at the level of the body centre of mass is not an exact recreation of inertial effects. However, we believe that by inducing similar mechanical requirements, this approach allows us to learn about the type of adaptations that are necessary to accelerate the body's centre of mass overground.

One potentially confounding issue when comparing acceleration data with constant-speed data is that all strides may not be at the same average speed. As can be seen from table 2, the average speed was not different between CS and ACC. Furthermore, this is an inherent advantage of using the normalized indexes and net work as metrics in this scenario, as they should not be biased by changes in speed.

Finally, this study only explored one acceleration condition. It would be interesting to explore whether the adjustments seen to induce acceleration are made in a way that is proportional to acceleration magnitude, such that control signals might be easily adjusted to recreate a similar effect in an assistive device.

## 5. Conclusion

To summarize, we found that only for walking and only for the ankle joint was there a fundamental shift in primary functional index to produce acceleration. Thus, this is probably the only joint that, when mimicked by man-made devices, may require additional components to cope with providing acceleration. Other joints probably only require adjustments in their control schemes to provide accelerative walking and running gaits. Further work is needed to examine how adjustments for acceleration change as a function of acceleration magnitude and if muscle-level mechanisms reveal more than examining joint-level data.

## Supplementary Material

Supplement 1. Individual participant time normalised data for ankle, knee and hip joint angles and moments during all experimental conditions

## Data accessibility

The electronic supplementary material for this article contains a spreadsheet of time-normalized joint angle and moment data for each participant (individual participant mean).
